# Effects of Latent Toxoplasmosis on Autoimmune Thyroid Diseases in Pregnancy

**DOI:** 10.1371/journal.pone.0110878

**Published:** 2014-10-28

**Authors:** Šárka Kaňková, Lucie Procházková, Jaroslav Flegr, Pavel Calda, Drahomíra Springer, Eliška Potluková

**Affiliations:** 1 Department of Philosophy and History of Science, Faculty of Science, Charles University, Prague, Czech Republic; 2 Department of Obstetrics and Gynaecology, General University Hospital and First Faculty of Medicine, Charles University, Prague, Czech Republic; 3 Institute of Clinical Biochemistry and Laboratory Diagnostics, General University Hospital and First Faculty of Medicine, Charles University, Prague, Czech Republic; 4 Third Department of Medicine, General University Hospital and First Faculty of Medicine, Charles University, Prague, Czech Republic; Johns Hopkins Bloomberg School of Public Health, United States of America

## Abstract

**Background:**

Toxoplasmosis, one of the most common zoonotic diseases worldwide, can induce various hormonal and behavioural alterations in infected hosts, and its most common form, latent toxoplasmosis, influences the course of pregnancy. Autoimmune thyroid diseases (AITD) belong to the well-defined risk factors for adverse pregnancy outcomes. The aim of this study was to investigate whether there is a link between latent toxoplasmosis and maternal AITD in pregnancy.

**Methods:**

Cross-sectional study in 1248 consecutive pregnant women in the 9–12^th^ gestational weeks. Serum thyroid-stimulating hormone (TSH), thyroperoxidase antibodies (TPOAb), and free thyroxine (FT4) were assessed by chemiluminescence; the *Toxoplasma* status was detected by the complement fixation test (CFT) and anti-*Toxoplasma* IgG enzyme-linked immunosorbent assay (ELISA).

**Results:**

Overall, 22.5% of the women were positive for latent toxoplasmosis and 14.7% were screened positive for AITD. Women with latent toxoplasmosis had more often highly elevated TPOAb than the *Toxoplasma*-negative ones (*p* = 0.004), and latent toxoplasmosis was associated with decrease in serum TSH levels (*p* = 0.049). Moreover, we found a positive correlation between FT4 and the index of positivity for anti-*Toxoplasm*a IgG antibodies (*p* = 0.033), which was even stronger in the TPOAb-positive *Toxoplasma*-positive women, (*p* = 0.014), as well as a positive correlation between FT4 and log_2_ CFT (*p* = 0.009).

**Conclusions:**

Latent toxoplasmosis was associated with a mild increase in thyroid hormone production in pregnancy. The observed *Toxoplasma*-associated changes in the parameters of AITD are mild and do not seem to be clinically relevant; however, they could provide new clues to the complex pathogenesis of autoimmune thyroid diseases.

## Introduction

The course of pregnancy is influenced by a number of environmental factors (e.g. infections), as well as endogenous factors in the mother. Often, it is difficult to identify the cause of an obstetric complication. Thyroid diseases are among the well defined risk factors for adverse pregnancy outcomes. Maternal autoimmune thyroid diseases (AITD) have been linked to infertility, spontaneous abortion, premature delivery, preeclampsia, caesarean section, and even foetal death [1].

In the general population, AITD are quite common. In our previous studies involving about 8500 pregnant women from the Czech Republic, we have found hypothyroidism (either manifest or subclinical) in more than 5% of these women and positivity for antibodies against thyroperoxidase (TPOAb) in 10% of the study cohort [2]–[4]. Such a high prevalence of AITD in pregnant women can be regarded as a serious public health issue.

The reasons for such a high prevalence of AITD in the population remain unclear, with the multifactorial aetiology being the most popular hypothesis. It is known that people with a genetic predisposition may develop AITD after an infectious disease [5], [6]. The molecular mimicry hypothesis suggests the resemblance between human thyroid autoantigens and molecular components of microorganisms is responsible for many autoimmune diseases including AITD [7], [8]. It has also been suggested that there is a link between AITD and the protozoan *Toxoplasma gondii* [9], [10]. In their study involving 1591 pregnant women, Wassermann et al. have found that *T. gondii* IgG seropositivity was the only significant infectious explanatory cofactor associated with the elevation of TPOAb in pregnancy. A smaller study on 414 pregnant women did not confirm this association, but its primary aim was to evaluate the association of toxoplasmosis and mood disturbances in pregnancy [11]. However, until now, no study has addressed the effect of toxoplasmosis on thyroid hormone levels in patients with AITD.

Toxoplasmosis is an endemic zoonosis caused by the parasitic protist *Toxoplasma gondii*, the most prevalent human parasite in developed countries. The prevalence of latent toxoplasmosis in the population ranges from 20% to 80% depending on various environmental and sociological factors, including the number of cats in the environment, latitude, moisture, hygienic standards, and kitchen habits [12]. Life-long latent toxoplasmosis is usually considered to pose no health threat to immunocompetent individuals; however, it is accompanied by specific changes in the psychomotor performance, behaviour, and personality profile [13]–[15]. A recent correlation study performed on a set of 88 countries has also shown that the prevalence of toxoplasmosis explains about 13% of the variation in the rates of congenital abnormalities between the countries [16].

Unlike acute toxoplasmosis in pregnancy, which has been described to damage significantly the foetal development [12], [17], latent toxoplasmosis seems to have no significant negative impact on the health of the offspring. However, latent toxoplasmosis leads to alterations in serum concentrations of testosterone both in men and mice [18], [19]. In addition to lower testosterone, the impairment of thyrotropin-releasing hormone (TRH) and thyroid-stimulating hormone (TSH) secretion as well as decreased serum thyroxine (T4) have been reported in *T. gondii*-infected mice [20], [21]. Latent toxoplasmosis is reportedly associated with higher concentration of cholesterol and LDL cholesterol in male schizophrenia patients [22]. A correlation study on a set of 88 countries has also shown a general positive association between prevalence of toxoplasmosis and incidence of endocrine disorders [16]. Moreover, it has been demonstrated that latent infection by *T. gondii* may lead to immune suppression both in mice and humans [23], [24].

Latent toxoplasmosis is thus a highly prevalent disease leading to alterations in the immune system and hormonal profile. A possible link between latent toxoplasmosis and AITD in pregnancy needs to be addressed. The goal of this study was to investigate whether or not there is an association between latent maternal toxoplasmosis, detected by immunological tests, and AITD in pregnant women diagnosed based on serum levels of TPOAb and thyroid hormones in the first trimester of pregnancy.

## Subjects and Methods

The study was designed as a retrospective cross-sectional study and was performed in cooperation of three clinical settings of the General University Hospital in Prague (Dept. of Obstetrics and Gynecology, Institute of Clinical Biochemistry and Laboratory Diagnostics, and the Third Department of Medicine) and the Department of Philosophy and History of Science, Faculty of Science, Charles University in Prague.

### Subjects

A total of 1250 consecutive women screened for AITD in 2008 and 2009 in the General University Hospital in Prague were included in the study. They were examined within an experimental universal screening programme for AITD conducted in 2006–2009 (gestational wks 9–12) [2]. The screening was focused on autoantibodies against thyroperoxidase (TPOAb), thyroid stimulating hormone (TSH), and free thyroxine (FT4). FT4 was assessed only in women with pathological TSH and/or positive for TPOAb. The laboratory assessment was performed in a single laboratory at the Institute of Clinical Biochemistry and Laboratory Diagnostics.

The AITD screening was carried out as part of the routine screening for chromosomal abnormalities (i.e. serum free beta-hCG and PAPP-A) in the 9–12^th^ gestational weeks in all consecutive study subjects regardless of their medical history or symptoms. Serum samples collected at the time of screening, were frozen and stored at −70°C for later evaluation. They were used for retrospective measurement of antibodies against *T. gondii.*

Moreover, we assessed the history of the women and the clinical outcome of the pregnancy using data extraction from the hospital database.

During the whole time of the project, we worked with an anonymous data set; the key for the identification of the individual participants was only accessible to the main authors of the study. All participating women signed an informed consent form. The study was approved by the Ethical Committee of the General University Hospital and the First Medical Faculty of the Charles University in Prague.

### Serological analysis and reference intervals

#### a) Screening for autoimmune thyroid disease

Serum samples were collected at inclusion after an overnight fast. The analysis of TSH, TPOAb, and FT4 was performed within eight hours. The rest of the serum sample was stored in aliquots frozen at −20°C until further use (screening for toxoplasmosis). TPOAb, TSH, and FT4 were assayed on the ADVIA Centaur Analyzer (Siemens Healthcare Diagnostics Inc., Tarrytown, NY) with chemiluminometric detection. TSH was determined using a direct sandwich chemiluminescence immunoassay and anti-TPO and FT4 were measured using a competitive chemiluminescence immunoassay.

The reference interval for TSH in the first trimester of pregnancy was set at 0.06–3.67 mIU/l [2], the upper limit for TPOAb at 143 kU/l, and the reference interval for FT4 at 9.8–23.1 pmol/l. A positive screening result was thus defined as TSH below 0.06 or above 3.67 mU/l and/or TPOAb above 143 kU/l. The upper limit of the detection range for TPOAb was 10000 kU/l when using automated sample dilution.

#### b) Immunological tests for toxoplasmosis

The complement-fixation test (CFT) determines the overall levels of IgM and IgG antibodies of particular specificity. This method has been used as a reference method in the Czech Republic in the last 50 years, which makes possible an analysis of the time trends in the seroprevalence of toxoplasmosis. Enzyme-Linked Immunosorbent Assays (ELISA) (IgG ELISA: SEVAC, Prague, IgM ELISA: TestLine, Brno) were used as a standard method. Using ELISA, individual classes of antibodies (IgG, IgM) were determined in order to discriminate between the acute and latent phase of the infection. Positivity for toxoplasmosis was defined as a CFT titre of 1∶8 and more, together with an index of positivity (IP) of >1.2 for IgG ELISA antibodies against *Toxoplasma*. The sera were retested when the results were ambiguous or when the results of two tests were in contradiction.

### Statistics

The Statistica 9.0 software was used for all statistical analyses. The influence of the binary variable latent toxoplasmosis (positive/negative) on the probability of a positive screening result was evaluated using the method of contingency tables. The following parameters were tested: any positive screening results, TSH elevation, TPOAb positivity (TPOAb^+^), TPOAb >500 kU/l, and combination of TSH elevation and TPOAb^+^.

The log-linear analysis of frequency tables was used for the analysis of a model with three binary variables: toxoplasmosis (Toxo) (positive/negative), TPOAb (TPOAb^+^/TPOAb^−^), and TSH (suppressed/normal/elevated).

Nonparametric tests, e.g. the Mann-Whitney test, were used to search for the influence of toxoplasmosis on the TSH, TPOAb, and FT4 levels. The influence of the levels of anti-*Toxoplasma-*antibodies (IP for IgG antibodies and CFT antibodies in a nonparametric test or log_2_ CFT antibodies in parametric linear regression) on the TPOAb, TSH, and FT4 levels was evaluated using Spearman correlation and that on the FT4 level was analysed using linear regression in the group of the *Toxoplasma*-positive women (FT4 levels had normal distribution).

The general linear model (GLM) was used to analyse the influence of the binary variable toxoplasmosis on serum free beta-hCG and PAPP-A, with the gestational age on the day of the blood sampling as a continuous variable and log free beta-hCG or log PAPP-A as dependent variables.

## Results

Overall, 1250 pregnant women, including 32 women who gave birth to twins, were enrolled in the study. Two women (one AITD negative and one AITD positive) were excluded due to suspected acute toxoplasmosis (tested positive for IgM antibodies against *T. gondii*). Our final set consisted of 1248 pregnant women; their mean age was 31 years (range 18–45 years). Of these, 183 (14.7%) were screened positive for AITD and 281 (22.5%) were diagnosed with latent toxoplasmosis. Basic characteristics of the women included in the study are shown in Table 1 and Table 2. *Toxoplasma* positivity frequencies in subgroups of pregnant women screened for thyroid disorders are shown in Table 3.

**Table 1:** Basic characteristics of women included in the study.

	Total	AITD+	AITD−	Toxo+	Toxo−	Toxo+ AITD+	Toxo+ AITD–	Toxo– AITD+	Toxo– AITD–
n	1248	183	1065	281	967	49	232	134	833
**Age (mean)**	31.0	31.3	30.9	31.7	30.8	32.5	31.5	30.8	30.8
	1.25 (0.01–37.57)	1.83 (0.01–37.57)	1.21 (0.07–3.65)	1.13 (0.01–21.75)	1.27 (0.01–37.57)	1.50 (0.01–21.75)	1.08 (0.08–3.65)	1.84 (0.01–37.57)	1,24 (0.07–3.64)
**TPOAb (kU/l)**	32 (0–10000)	279 (5–10000)	30 (0–142)	31.3 (0–10000)	32 (0–10000)	507 (11–10000)	29.75 (0–134)	247.5 (5–10000)	30 (0–142)
**TPOAb+**	10.2%	69.4%	-	12.5%	9.5%	71.4%	-	68.7%	-

AITD+/−: women screened positive/negative for autoimmune thyroid disease in pregnancy (TPOAb positivity and/or pathological TSH); Toxo+/−: women with/without latent toxoplasmosis. The women were included in the 9^th^–12^th^ gestational weeks. Except of age, values are expressed as medians and range.

**Table 2:** History of previous pregnancies and the outcome of the current pregnancy.

		Total (n)	Toxo+ AITD+ (n)	Toxo+ AITD– (n)	Toxo– AITD+ (n)	Toxo– AITD– (n)	OR
Total (n)		1248	49	232	134	833	1.31
**Number of previous pregnancies**	0	459	19	94	44	302	1.39
	1	340	13	57	39	231	1.35
	>1	125	4	27	13	81	0.92
	Not available	324	13	54	38	219	1.39
**Current Pregnancy Outcome**	Live birth	678	31	127	71	449	1.54
	Abortion or fetus loss	18	0	4	2	12	-
	Not available	552	18	101	61	372	1.09

AITD+/−: women screened positive/negative for autoimmune thyroid disease in pregnancy (TPOAb positivity and/or pathological TSH); Toxo+/−: women with/without latent toxoplasmosis. The last column shows OdsRatio reflecting increased (decreased) risk of autoimmune thyroid disease in *Toxoplasma*-infected subjects. No association was statistically significant.

**Table 3:** Frequencies of pregnant women screened for autoimmune thyroid disorders according to *Toxoplasma* positivity.

	Total	Negative in screening	Positive in screening	TPOAb	negative		TPOAb	positive	
TSH				suppressed	elevated	normal	suppressed	elevated	all values
***Toxo*** **–**	967	833	134	20	22	71	3	18	92
***Toxo*** **+**	281	232	49	10	4	27	1	7	35
**Total**	1248	1065	183	30	26	98	4	25	127

Toxo–: women without toxoplasmosis; Toxo+: women with latent toxoplasmosis. Positivity in screening for autoimmune thyroid disorders included either positivity of TPOAb and/or pathological TSH.

### Latent toxoplasmosis and thyroid autoimmunity

**Figure 1:**
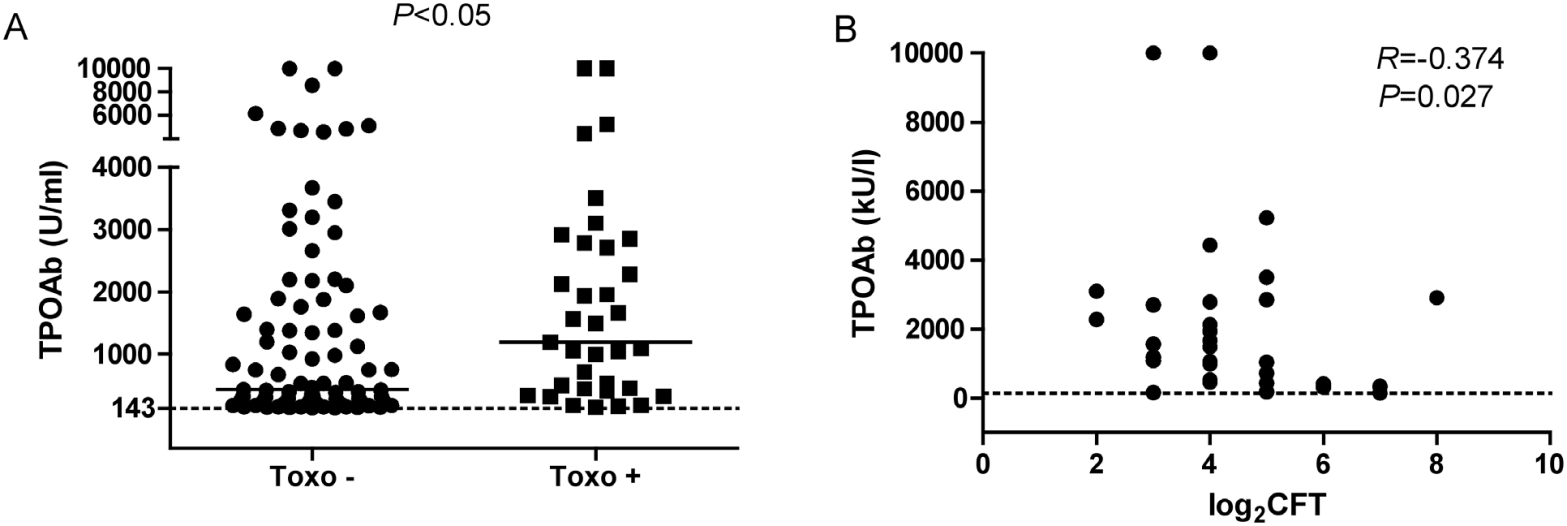
Association of latent toxoplasmosis and serum TPOAb levels in pregnant women (9–12th gest. wks). A: Serum TPOAb levels in *Toxoplasma*-negative vs. *Toxoplasma*-positive pregnant TPOAb-positive women. Black lines represent median values. (Mann-Whitney test). B: Negative correlation between log_2_ CFT (complement fixation test) *T. gondii* antibodies and serum TPOAb levels in the TPOAb-positive pregnant women with latent toxoplasmosis (Spearman correlation).

Although the overall comparison did not show an increased prevalence of TPOAb positivity in the *Toxoplasma*-positive women, we found an association of latent toxoplasmosis with high TPOAb levels. Among the 967 *Toxoplasma*-negative women, 134 (13.9%) were screened positive for AITD as compared to 49 (17.4%) of the 281 *Toxoplasma*-positive women (*p* = 0.135). Overall, TPOAb (>143 k/l) were present in 92 *Toxoplasma*-negative women (9.5%) and in 35 *Toxoplasma*-positive ones (12.5%) (*p* = 0.151). However, a subgroup analysis showed that the *Toxoplasma*-positive women were more frequently highly positive for TPOAb (>500 kU/l) than the *Toxoplasma*-negative ones: 25/281 (8.9%) vs. 43/967 (4.4%) (*χ^2^* = 8.37, *p* = 0.004). Moreover, latent toxoplasmosis in the TPOAb–positive pregnant women was associated with elevated TPOAb levels (*n* = 127, *Z* = 2.45, *p* = 0.014) (Fig. 1A). Although there was no correlation between serum TPOAb levels and the index of positivity for IgG antibodies against *T. gondii* (IP for IgG), we found a negative association between CFT antibody titres and TPOAb levels (*n* = 35, Spearman *R* = −0.37, *p* = 0.026) in the TPOAb–positive pregnant women with latent toxoplasmosis (Fig. 1B).

### Latent toxoplasmosis and thyroid hormones

**Figure 2:**
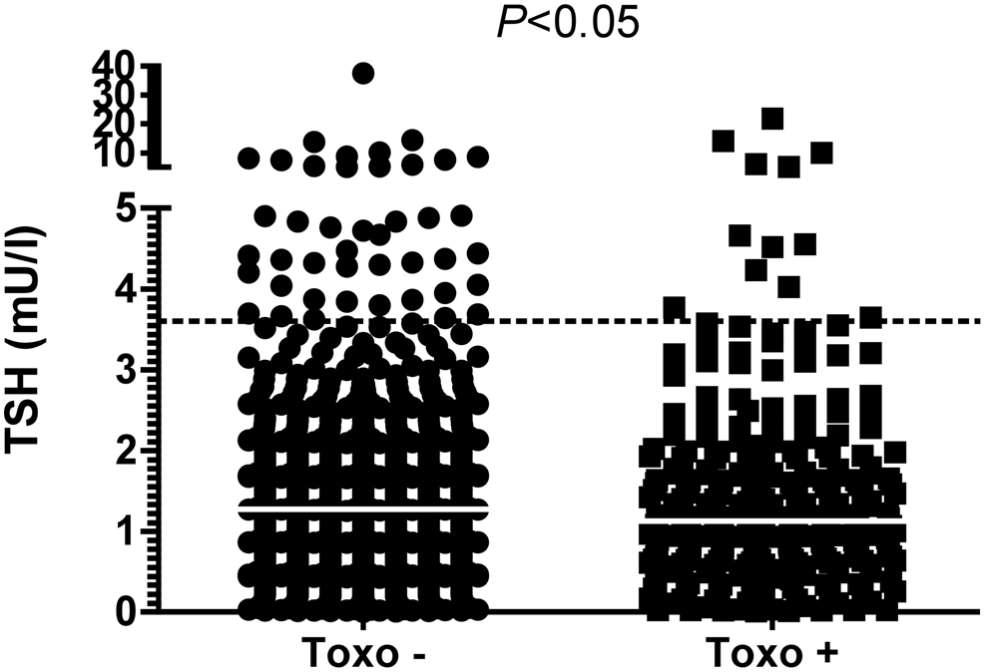
Comparison of serum TSH in pregnant women (9–12th gest. wks) with and without latent toxoplasmosis. The dotted lines represent the upper cut-off for normal values. White lines represent median values. Mann-Whitney test.

Latent toxoplasmosis was associated with a decrease in TSH levels. Women with latent toxoplasmosis had lower TSH levels than the *Toxoplasma*-negative ones (*Z* = 1.97, *p* = 0.049; median 1.13 vs. 1.27 in the *Toxo*+ vs. *Toxo–* women; SD = 1.82) (Fig. 2). Decreased levels of TSH (<0.06 mIU/l) were detected in 23 *Toxoplasma*-negative women (2.5%) and in 11 *Toxoplasma*-positive ones (4.1%) (*p* = 0.166, chi-square test).

**Figure 3:**
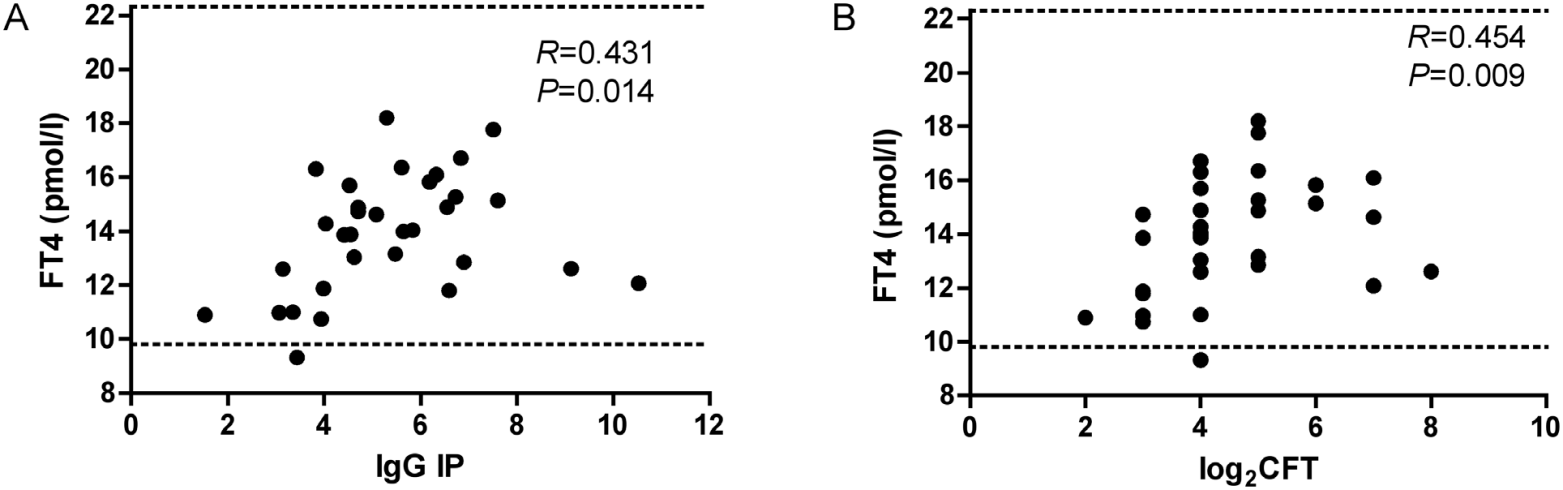
Relationship between free thyroxine (FT4) and positivity for *Toxoplasma* antibodies in TPOAb-positive pregnant women with latent toxoplasmosis. A: Correlation of free thyroxine (FT4) and index of positivity for anti-*Toxoplasma* IgG antibodies (IP for IgG). B: Correlation of FT4 and logarithmic values of the complement fixation test (CFT) antibodies against *T. gondii* (Spearman correlation). Dotted lines represent the reference range for FT4.

The IP for IgG in the *Toxoplasma*-positive women positively correlated with the FT4 level (*n* = 76, *F* = 4.71, *R* = 0.24, *p* = 0.033). This correlation was even stronger in the TPOAb–positive pregnant women with latent toxoplasmosis (*n* = 32, Spearman *R* = 0.43 *p* = 0.014; Fig. 3A). In this subgroup, also a positive correlation existed between CFT antibody titres and FT4 levels (*n* = 32, Spearman *R* = 0.45, *p* = 0.009; Fig. 3B).

The statistical analysis did not show any correlation either between the IP for IgG or between the CFT antibody titres and TSH or TPOAb in the *Toxoplasma*-positive women.

In our data set, no association was observed between latent toxoplasmosis and hypothyroidism in pregnancy. Elevated TSH levels (>3.67 mIU/l) were found in 40 (4.2%) *Toxoplasma*-negative women and in 11 (4.1%) *Toxoplasma*-positive women (Table 1). A model with three variables: latent toxoplasmosis (positive vs. negative), TPOAb (TPOAb^+^ v.s. TPOAb^−^), and TSH (suppressed/normal/elevated) did not show any significant association between the binary variables analysed (*p* = 0.398).

### Latent toxoplasmosis and serum free beta-hCG and PAPP-A

The statistical analysis did not show any significant association of latent toxoplasmosis either with the serum free beta-hCG (*p* = 0.808) or the serum PAPP-A (*p* = 0.465) in the pregnant women in the 9–12^th^ gestational weeks.

## Discussion

In this study, we assess the prevalence of latent toxoplasmosis in pregnancy and analyse its possible association with autoimmune thyroid diseases. The study cohort were consecutive women in the first trimester of pregnancy (9–12^th^ gestational weeks) screened for AITD by measurement of TSH, TPOAb, and FT4. The presence of latent toxoplasmosis was defined as a positive complement-fixation test and anti-*Toxoplasma* IgG positivity in ELISA (women with IgM positivity were excluded).

Of the 1248 pregnant women, 22.5% were screened positive for latent toxoplasmosis and 14.7% for AITD. Although we could not detect any strong effect of latent toxoplasmosis on the presence of AITD, we found that women with latent toxoplasmosis had more often highly elevated TPOAb than the *Toxoplasma*-negative ones. Furthermore, latent toxoplasmosis had a week stimulatory effect on serum levels of thyroid hormones.

The prevalence of latent toxoplasmosis in our cohort is in line with the data of the Centers for Disease Control and Prevention (CDC) reporting that 22.5% of the North American population older than 12 years of age are positive for *Toxoplasma* [25], but it is slightly lower than the rate, i.e. 26.7%, found by Wasserman et al. in a large mixed group of pregnant women residing both in rural and urban areas [10]. The pregnant women included in our study were mostly residents of the capital city and only a few of them lived in rural areas. It is highly probable that the prevalence of latent toxoplasmosis would be higher if more rural residents were included. Toxoplasmosis has been reported to be more prevalent in rural areas in many countries, including the Czech Republic [26], probably due to the more frequent contact with *Toxoplasma* oocytes excreted by cats.

In contrast to Tozzoli et al., who found a 65.5% prevalence of latent toxoplasmosis among patients with AITD [27], only 27.1% of the 127 TPOAb-positive pregnant women were positive for latent toxoplasmosis in our study. Tozzoli analysed a group of 120 AITD patients from different areas of north-western Italy. Although the numbers of AITD patients analysed are similar, there are some potentially important differences between these two groups. Our cohort consisted of pregnant women with mostly mild forms of Hashimoto’s thyroiditis (HT) including subclinical cases, with no identified Graves’ disease (GD) cases; and it was relatively homogeneous, consisting mostly of capital city residents. Tozzoli et al. have not characterised their AITD group in detail, but it appeared to be more heterogeneous with a large proportion of Graves’ disease cases (there was no difference in the prevalence of toxoplasmosis in HT and GD, however).

The results of our study differ also from the data reported by Wassermann et al. They have found a strong association between TPOAb and antibodies against *T. gondii* in a comparably large group of pregnant women analysed in the second half of pregnancy [10]. In our study, there was a significant association between latent toxoplasmosis and TPOAb levels only in the TPOAb-positive women. Interestingly, we found a negative correlation between the CFT titres of anti-*Toxoplasma* antibodies and TPOAb levels in these women. It is known that the CFT titres decrease with the duration of the latent infection by the protozoan [28], therefore, we can speculate that the production of the TPOAb increases with the time from the infection by *T. gondii.* This suggests that the observed phenomenon represents a cumulative effect of latent toxoplasmosis, rather than a fade away effect of past acute toxoplasmosis.

Different results obtained in our study and works by others [10], [27] concerning the percentages of women with positive autoantibody tests could be explain by geographical differences in autoantibodies generation patterns [29].

Our study is the first to address the effect of latent toxoplasmosis on thyroid hormones production. We demonstrate that the infection is not associated with hypothyroidism, as could be expected, as a result of the promotion of thyroid autoimmunity, but that it is associated with a slight decrease in TSH and an increase in FT4 (as observed only in TPOAb-positive/*Toxoplasma*-positive women). Moreover, the correlation analysis showed that women with a higher index of positivity for *T. gondii* IgG antibodies, as well as with a higher CFT antibody titre, had higher concentrations of FT4. These concentrations remain, however, at subclinical levels. Furthermore, we could exclude an effect of latent toxoplasmosis on the serum concentration of HCG, which is known to have a TSH-like effect in pregnancy leading to transitory gestational hyperthyroidism [30]. Our data thus indicate that latent toxoplasmosis *per se* might have a moderate stimulatory effect on thyroid hormone production in pregnancy. We can just speculate about the mechanisms responsible for the observed phenomena. During early pregnancy, an initial shift from Th1 to Th2 immune reactions occurs as part of the mechanisms of maternal immune tolerance of the foetus, as a semi-allograft [31]. This may also lead to a reactivation of *Toxoplasma* infection, because under normal conditions, this intracellular parasite is controlled mostly by Th1 immune reactions [32], [33]. It is possible that this Th1 to Th2 shift could also be behind a partial escape of *T. gondii* from immunological control with subsequent activation of autoimmune mechanisms that advance the progression of pre-existing thyroiditis with a transient release of thyroid hormones. Thus, it would be interesting to analyse the values of maternal thyroid parameters in relationship to the *Toxoplasma* positivity in the second half of pregnancy, when the Th1 response shifts back to Th2, and also after delivery.

A possible role of the NK cells in the interaction between *Toxoplasma* and thyroid autoimmunity should be mentioned. As Solerte et al. have shown, important alterations in the NK cell function are associated with the autoimmune thyroid disorders and these changes are probably related to the onset and progression of the autoimmune mechanism [34]. NK cells are important mediators of the immune response against *Toxoplasma* via a robust IFN-γ-mediated effect that limits parasite replication and allows for parasite clearance [35]. A defect of the NK cell activity in autoimmune thyroiditis could thus eventually result in a higher risk of reinfection by *T. gondii* during pregnancy [36].

Also, fetal microchimeric cells have been more frequently found in the thyroid gland of women with Hashimoto’s thyroiditis and Graves’ disease compared to those women without thyroid autoimmunity [37], [38]. It was suggested that *Toxoplasma*-containing cells of fetal origin could disseminate the parasite in the maternal organism [39]. It was reported recently that *Toxoplasma* increases migration activity of infected leukocytes, which could help spread infection in various tissues of the host body [40].

Our study has several limitations. Most importantly, the numbers of pregnant women with thyroid dysfunction (hypo−/hyperthyroidism) are too low to perform a reliable statistical analysis of the thyroid hormonal secretion with regard to the *Toxoplasma* status. Moreover, we are lacking the follow-up data on the thyroid hormone levels, which would improve the understanding of the mechanism by which *Toxoplasma* influences thyroid autoimmunity/function.

In conclusion, our study provides evidence for the existence of an association between latent toxoplasmosis and thyroid autoimmunity and thyroid function in pregnancy. This association does not seem to be clinically relevant; however, they could provide new clues to the understanding of the complex pathogenesis of autoimmune thyroid diseases.
